# Reduced cerebellar cortical thickness in World Trade Center responders with cognitive impairment

**DOI:** 10.1038/s41398-022-01873-6

**Published:** 2022-03-16

**Authors:** Sean A. P. Clouston, Minos Kritikos, Chuan Huang, Pei-Fen Kuan, Paul Vaska, Alison C. Pellecchia, Stephanie Santiago-Michels, Melissa A. Carr, Sam Gandy, Mary Sano, Evelyn J. Bromet, Roberto G. Lucchini, Benjamin J. Luft

**Affiliations:** 1 Department of Family, Population, and Preventive Medicine, Renaissance School of Medicine at Stony Brook, Stony Brook, NY USA; 2 Program in Public Health, Renaissance School of Medicine at Stony Brook, Stony Brook, NY USA; 3 Department of Radiology, Renaissance School of Medicine at Stony Brook, Stony Brook, NY USA; 4 Department of Applied Mathematics, Stony Brook University, Stony Brook, NY USA; 5 Department of Biomedical Engineering, Stony Brook University, Stony Brook, NY USA; 6 Department of Medicine, Renaissance School of Medicine at Stony Brook, Stony Brook, NY USA; 7 Center for Cognitive Health and NFL Neurological Care, Department of Neurology, Icahn School of Medicine at Mount Sinai, New York, NY USA; 8 Mount Sinai Alzheimer’s Disease Research Center, Department of Psychiatry, Icahn School of Medicine at Mount Sinai, New York, NY USA; 9 Department of Psychiatry, Renaissance School of Medicine at Stony Brook, Stony Brook, NY USA; 10 Department of Environmental Health Sciences, Robert Stempel College of Public Health, Florida International University, Miami, FL USA; 11 Director of the World Trade Center Health and Wellness Program, Department of Medicine, Renaissance School of Medicine at Stony Brook, Stony Brook, NY USA

**Keywords:** Diagnostic markers, Learning and memory

## Abstract

Prior research has demonstrated high levels of cognitive and physical functional impairments in World Trade Center (WTC) responders. A follow-up neuroimaging study identified changes to white matter connectivity within the cerebellum in responders with cognitive impairment (CI). In the first study to examine cerebellar cortical thickness in WTC responders with CI, we fielded a structural magnetic resonance imaging protocol. WTC responders (*N* = 99) participated in a structural magnetic resonance imaging (MRI) study, of whom 48 had CI. Participants with CI did not differ demographically or by intracranial volume when compared to cognitively unimpaired participants. MRIs were processed using the CERES imaging pipeline; bilateral cortical thickness in 12 cerebellar lobules was reported. Analyses were completed comparing mean cerebellar cortical thickness across groups. Lobules were examined to determine the location and functional correlates of reduced cerebellar cortical thickness. Multivariable-adjusted analyses accounted for the false discovery rate. Mean cerebellar cortical thickness was reduced by 0.17 mm in responders with CI. Decrements in cerebellar cortical thickness were symmetric and located in the Cerebellar Crus (I and II), and in Lobules IV, VI, VIIb, VIIIa, VIIIb, and IX. Cerebellar cortical thickness was associated with episodic memory, response speed, and tandem balance. WTC responders with CI had evidence of reduced cerebellar cortical thickness that was present across lobules in a pattern unique to this cohort.

## Introduction

The cerebellum is central to appropriating motor system operations, and functions by relaying information between cerebellar motor neurons with cortical neurons throughout the brain’s cognitive and sensory networks. Beyond its sensorimotor and vestibular control, prior research has shown the cerebellum to be crucial to cognitive function [1], whereby posterior lobe lesions have been demonstrated to result in deficits in executive function, visuospatial processing, linguistic skills, affective processing, and changes in obsessive and impulsive behaviors [2, 3]. The cerebellum’s defining feature is that it performs different functional processes within highly localized components, with strong associations noted between the cerebellar vermis and the limbic system, regulating emotion, executive function, language, and working memory [4]. Since the cerebellum acts as a relay station to help coordinate motor functioning with cognitive, sensory, and emotional functioning [5], cerebellar atrophy might contribute to reduced coordination between systems and may help explain concurrent loss across domains of cognition and motor functioning [6]. Cerebellar atrophy presents in a localized manner that might enable researchers to differentiate between common neurodegenerative diseases, such as Alzheimer’s disease (AD), frontotemporal dementia (FTD), amyotrophic lateral sclerosis (ALS), multiple system atrophy (MSA), and progressive supranuclear palsy (PSP), while enabling the exclusion of other diseases such as Huntington’s (HD) and Parkinson’s diseases (PD) [7].

The potential for cerebellar gray matter atrophy to help with differential diagnosis is relatively unique and may help us to understand the etiology of novel diseases in exposed populations where symptoms are only beginning to emerge. For example, recent work in World Trade Center (WTC) responders suggests that a novel disease might be present [8]. Indeed, this work has identified symptoms consistent with generalized cognitive impairment (CI) with losses focused on response speed, memory, and throughput [9]. Similarly, physical functional impairments are emerging alongside post-traumatic stress disorder (PTSD) and CI in WTC responders [10–12]. Coupled with those changes we have found evidence of cortical [13] and hippocampal atrophy [14], as well as the presence of plasma-based measures of Amyloid, Tau, and Neurodegenerative involvement [15]. As a result, a recent review concluded that WTC responders may be at heightened risk of developing Alzheimer’s disease or related dementia (ADRD) [16].

A recent study of cerebral connectivity in WTC responders with mild cognitive impairment (MCI) identified widespread connectivity changes in the cerebellar white matter that might indicate early cerebellar atrophy [17]. These findings suggest that responders are either exhibiting a form of ADRD or have developed a novel WTC exposure-related condition. The objective of the present study was to examine these alternative hypotheses to shed light on whether WTC responders are at increased risk for a known neurodegenerative condition or are instead experiencing an undetermined encephalopathy associated with their 9/11 exposures. Therefore, we proposed that examining the location of cerebellar gray matter atrophy may help elucidate and even differentiate between neuropathological conditions [7]. We hypothesized that (1) WTC responders with CI would have evidence of greater cerebellar atrophy when compared to cognitively unimpaired responders, (2) that cerebellar cortical thickness would be associated with cognitive and physical functioning, and (3) the pattern of reduced cerebellar cortical thickness might match other common neurodegenerative conditions or, if not, might present as a novel WTC exposure-related condition.

## Methods

### Participants

The present study used a novel routine to analyze data that have been previously described [18]. Briefly, from 2017–2019 we recruited 99 responders aged 44–65 from a monitoring program to participate in a structural neuroimaging study. Responders were sampled to fill four matched groups including individuals with cognitive impairment (*N* = 48). The demographic characteristics of these two groups are shown in Table 1. While we also matched groups based on PTSD status, because we have previously found no significant association between PTSD and cortical thickness in the cerebrum [18], no reductions in cortical thickness were expected here and sensitivity analyses supported this result justifying the focus on CI in this study.

**Table 1:** Sample characteristics.

	Whole sample (*N* = 99)		Cognitively unimpaired (*N* = 51)		Cognitively impaired (*N* = 48)		
	%		%		%		*P*-value
Female	21.2%		19.6%		22.9%		0.687
University degree	29.3%		33.3%		25.0%		0.363
Law enforcement	73.7%		80.4%		66.7%		0.121
Race/ethnicity							
White	73.7%		84.3%		62.5%		0.104
Hispanic	12.1%		7.8%		16.7%		
Black	10.1%		5.9%		14.6%		
Other	4.0%		2.0%		6.3%		
Post-traumatic stress disorder	47.5%		47.1%		47.9%		0.932

	Mean	SD	Mean	SD	Mean	SD	*P*-value
Age, years	56.37	5.19	56.37	4.59	56.36	5.81	0.993
SPPB Score	10.78	2.03	11.02	1.78	10.51	2.25	0.057
Gait Score	3.81	0.59	3.86	0.49	3.74	0.67	0.410
Mean walking speed, m/s	0.94	0.20	0.99	0.19	0.89	0.20	0.023
Chair Rise Score	3.17	1.12	3.31	1.10	3.02	1.13	0.078
Mean chair-rise speed, r/s	0.53	0.18	0.55	0.17	0.51	0.18	0.287
Balance Score	3.80	0.77	3.84	0.70	3.74	0.85	0.402
Maximal handgrip, lbs	56.58	17.46	58.27	16.35	54.77	18.58	0.362
Episodic memory	0.64	0.17	0.72	0.19	0.56	0.10	2.22E-06
Intraindividual response variability	0.10	0.04	0.09	0.03	0.12	0.04	3.04E-05
Response speed, r/ds	0.76	0.09	0.79	0.07	0.72	0.09	1.01E-04
Visuospatial learning	75.08	47.36	57.08	15.42	95.04	61.21	1.55E-04
Visuospatial memory	13.28	9.09	9.47	4.33	17.50	10.99	2.03E-05
Processing speed, r/ds	0.63	0.05	0.65	0.04	0.61	0.06	2.67E-04
Attention	1.36	0.20	1.43	0.14	1.28	0.22	1.71E-04
Visual memory	0.95	0.12	0.99	0.12	0.91	0.10	3.27E-04
Throughput	0.32	0.04	0.33	0.04	0.30	0.03	3.55E-05
Intracranial volume, cm^3^	1578.50	147.00	1583.26	128.25	1573.44	165.86	0.744
Cerebellar cortical thickness, mm	5.00	0.29	5.08	0.00	4.91	0.00	0.002
Cerebellar asymmetry, %	−0.32	1.33	−0.43	0.27	−0.20	0.29	0.406

*P*-values determined using Welch’s *t*-test and tests of proportions to compare CU to CI group.

*CU* cognitively unimpaired, *CI* cognitively impaired, *SPBB* Short Physical Performance Battery, *IRV* intraindividual response variability, *SD* standard deviation, *cm* centimeter, *mm* millimeter, *lbs* pounds.

### Image acquisition

Participants underwent magnetic resonance imaging using a Siemens 3 T Biograph mMR (software V.VE11P) with a 20-channel head/neck coil. A structural T1-weighted magnetization prepared rapid gradient echo (T1-MPRAGE) protocol (TR = 1900s, TE = 2.49 ms, TI = 900 ms; Flip Angle = 9°, voxel resolution = 0.89 × 0.89 × 0.89 mm) was acquired to examine structural correlates of cognitive impairment.

### Cerebellar quantification

**Fig. 1:**
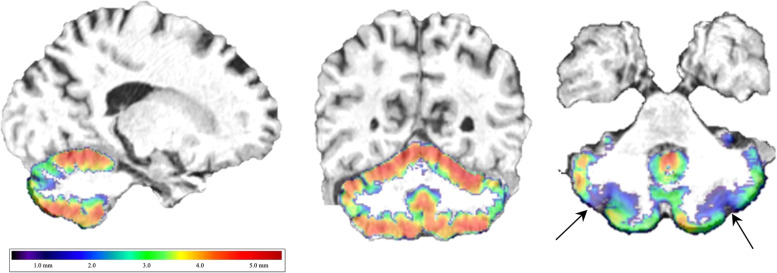
Cerebellar cortical thickness map. Example of sagittal and coronal cerebellar cortical thickness estimate maps employed in this present study using a rainbow colormap to visually identify regional asymmetry in cortical thickness, such as in the cerebellar crus in this example of a responder with Cognitive Impairment.

For cerebellar analyses, we used the CERES automated pipeline to segment the cerebellar lobule into 13 lobules including the two cerebellar crus [19]. The CERES automated pipeline was selected because prior comparative analyses have identified that this pipeline has particularly high replicability [20] coupled with relatively low coefficients of variation [21] when compared to other methods. This pipeline automatically provided bilateral and unilateral estimates of gray matter volume (expressed in cubic centimeters [cm^3^] and percent intracranial volume [ICV]) and cortical thickness, as shown in Fig. 1. However, because of a high degree of association between ICV and volumetric measures in general, we focused on cortical thickness as an accurate reflection of risk for cortical atrophy that minimized the influence of ICV. Measures were provided in the whole cerebellum and across 12 subregions (Lobules I and II were combined into a shared region by the parcellation program), including each of ten lobules and two subregions within the cerebellar crus. Because left/right asymmetry in cerebellar atrophy is common in several neurodegenerative diseases including AD [7], the degree of lobular asymmetry was recorded.

### Inclusion/exclusion criteria

Detailed exclusion criteria included the history of psychosis, uncontrolled diabetes, heart attack within the past year, diagnoses for brain cancer, Alzheimer’s disease, general dementia, major stroke, epilepsy, multiple sclerosis, and Parkinson’s disease, or head injuries during the WTC response or during military deployment; current renal or liver disease; or current use of cognitively active medications as well as those for hepatitis or liver disease. Subjects also satisfied eligibility criteria for MRI scanning including body mass index ≤40, no known pregnancy or claustrophobia, and no known metal implants/shrapnel not deemed MRI- safe.

### Clinical measures

CI was defined algorithmically following standard criteria [22]. All imaged responders with CI fit diagnostic criteria consistent with multidomain CI and mild limitations in numeracy, visuospatial functioning, and orientation consistent with possible dementia. Global cognitive status was objectively assessed using the Montreal Cognitive Assessment (MoCA) [23], with a conservative cutoff score (MoCA ≤ 20) used to identify cognitive impairment [18]. Cognitively unimpaired (CU) responders scored within the normal range (MoCA ≥ 26).

### Cognitive functioning

Cognitive functioning across several domains was assessed with the CogState battery [24] in all patients. The Cogstate battery consisted of repeated computer-administered game-like tasks [25–27]. Five tasks were utilized to measure cognitive dysfunction across domains. From these tasks, we retrieved the following measures: throughput (One-card learning accuracy divided by testing speed), visual memory (One-card learning), episodic memory (Continuous Paired Associate Learning), visuospatial learning (Groton Maze learning test), visuospatial recall (Groton Maze learning test delayed recall), intraindividual item-response variability (Detection response variability), reaction speed (Detection responses per second), and processing speed (Identification responses per second). While data were available for all participants on three of these tests, two study participants did not complete the Groton Maze and Continuous Paired Associate tests for this study.

### Physical functioning

Physical functional limitations were measured using the Short Physical Performance Battery (SPPB) [28]. Trained research staff followed standard methods of administration. The domains of the SPPB analyzed here are chair-rise speed (rises/second), walking speed (meters/second), and balance functioning (any difficulties in any task). The presence or absence of functional limitations was reported as an overall score, with a total score of ≤9 from a maximum of 12 indicating one or more mobility limitations [29]. Additionally, maximal handgrip strength (lbs.) was measured using Vernier™ computer-assisted handgrip dynamometers. One responder refused to complete the physical performance test, and two other responders who could not complete the SPPB were excluded from subdomain analyses but were scored according to SPPB standards.

### WTC exposure duration

WTC exposure duration, measured as the length of time working in the pit or on the pile, has previously been associated with CI [30, 31]. This study examined cumulative time working at the WTC sites, as expressed in months, and collected at enrollment into the parent study to indicate WTC exposure severity.

### Additional sample characteristics

As reported in Table 1, we first compared the CI versus CU groups on age, sex, education, occupation, and race. The presence of post-traumatic stress disorder and severity of different symptoms types were determined by the Structured Clinical Interview for the DSM-IV (SCID-IV) [32].

### Multi-region risk scores

Drawing on results from a prior review [7], we calculated multi-region risk scores for MSA, ALS, FTD, PD, HD, AD, and PSP by combining information from unilateral regions of interest as noted in Table 3. Multiregional scores were calculated by taking averages of standardized cerebellar thicknesses in each region for diseases. The predictive power for these regions was then compared, however, because each regional score was associated (Pearson’s R ranged from 0.60–0.95, *p* < 0.001 in all cases) we also calculated orthogonalized multiregional scores using the overall mean cortical thickness to capture generalized cerebellar degeneration.

### Statistical analysis

We began by showing sample characteristics using percentages for categorical variables and means and standard deviations for continuous ones both for the whole sample and stratified into CI and CU responders. Welch’s *t*-tests and tests of proportions were used to examine differences between these two groups. To help readers visualize cerebellum placement and identify potential changes in the cerebellum, an example of the image colored using the rainbow spectrum colormap with a cortical thickness mapping was provided (see Fig. 1).

The analytic plan began at the global level to examine the evidence for reduced cerebellar cortical thickness before then focusing on bilateral and then unilateral subregions to help better determine levels of reduced cerebellar cortical thickness in responders with CI. All differences were examined both without adjustment for gender, and then again with adjustment. Because post-hoc model adjustment is not recommended in studies using a matching protocol [33], multivariable-adjusted models were not adjusted for matching criteria in analyses except in the case of gender and race/ethnicity, where larger and potentially influential differences were observed. Subgroup analyses were also used to examine results for men and women separately to examine the potential for sex-based differences to emerge. Next, we examined correlations between subdomain measures of cognition and of physical functioning. A representative scatter plot with best-fitting linear association stratified by gender were used to show the overall differences between cerebellar cortical thickness in women and men. Multivariable-adjusted results examining associations between cerebellar volume measures and both cognitive and physical subdomain measures while adjusting for sex and race/ethnicity were completed using linear modeling. Results are provided in text and in heat maps.

Statistical analyses relied on a two-tailed α = 0.05 to determine statistical significance. Levels of statistical significance were adjusted for the false discovery rate [34]. We tested assumptions implicit in each model. Statistical analyses were completed using Stata 17/MP [StataCorp].

Statistical Power for this study was set to identify differences in cortical thickness between cognitively unimpaired and responders with mild dementia [35]. The sample size was set to identify a difference exceeding 0.6 standard deviations between the two groups.

Sensitivity analyses were completed to examine WTC-specific exposures including PTSD and exposure duration. To examine the role of PTSD, we also examined differences between responders with and without PTSD using Welch’s *t*-tests. Analyses with PTSD status, PTSD symptom subdomains, cognitive domains, and exposure duration also used linear regression both with and without adjusting for participant sex and race/ethnicity. Finally, since prior research has suggested alcohol intake may cause cerebellar atrophy, we examined the need to adjust for alcohol intake. These analyses showed no association in bivariate analyses between cerebellar either CI (*p* = 0.696) or cortical thickness (*p* = 0.818) and alcohol intake measured using the Alcohol Use Disorders Identification Test (instrumentalized either using the raw score or using standard cutoffs (≥10)). Since multivariable-adjusted models similarly showed no strong results, we did not report these analyses in this study.

### Ethics

The Institutional Review Boards at Stony Brook University and the Icahn School of Medicine at Mount Sinai approved all study procedures. All participants provided written informed consent at study enrollment and at the neuroimaging appointment.

## Results

The sample characteristics are shown in Table 1. The average responder was in their mid-fifties, and the majority were male. Cerebellar cortical thickness differed by cognitive status, but cerebellar white matter volume, intracranial volume, and cortical asymmetry did not differ between groups. The CI group had significantly worse episodic memory, higher intraindividual response variability, slower response and processing speed, worse visuospatial learning and memory, lower overall attention, poorer visual memory, and lower throughput. The CI group also had slower walking speed and lower mean cerebellar cortical thickness.

**Fig. 2:**
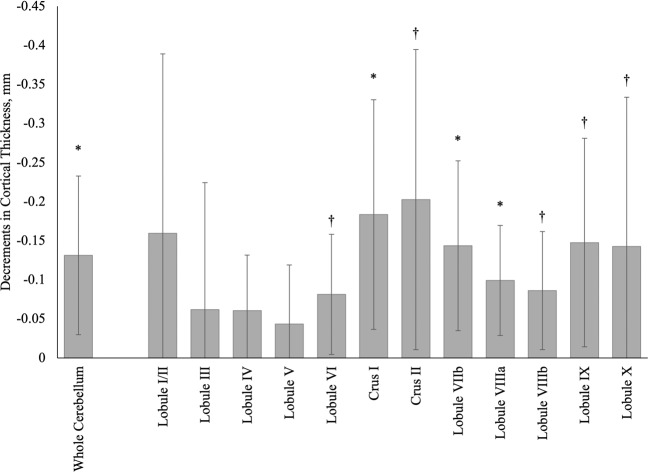
Reductions in mean bilateral cortical thickness (mm) in the whole cerebellum and across 12 cerebellar regions of interest in World Trade Center responders with cognitive impairment (*n* = 48) when compared to WTC responders who were cognitively unimpaired (*n* = 51). Confidence intervals are shown using error bars. Reductions with confidence intervals not crossing the base line were nominally significant, while those marked with * survived adjustment for the false discovery rate.

In Fig. 2 we examined bilateral subregional differences and found that CI status was associated with the lower overall cerebellar cortical thickness (−0.17 mm). Atrophied regions, ranked from most to least prominent, included the cerebellar crus II (−0.28 mm) and crus I (−0.24 mm) as well as across lobules IX (−0.19 mm), VIIb (−0.18 mm), VIIIa (−0.12 mm), VIIIb (−0.11 mm), VI (−0.11 mm), and IV (−0.09 mm).

Further segmentation into unilateral subregions identified nominally significant cortical thickness reductions within regions from both the right and left hemispheres, predominantly in the right hemisphere (Table 2). Following adjustment for the false discovery rate, cortical thickness was associated with subregional reductions in the right (Crus I and II, and lobule VIIIa) and left (Crus II and in lobules VI, VIIb, VIIIa, and X) hemisphere.

**Table 2:** Asymmetric differences in cortical thickness by cerebellar subregion and whole cerebellum.

	Right				Left			
Region	B	SE	*P*	FDR	B	SE	*P*	FDR
Lobule I/II	−0.15	0.12	0.210		−0.17	0.12	0.165	
Lobule III	−0.02	0.08	0.834		−0.06	0.08	0.458	
Lobule IV	−0.07	0.04	0.069		−0.05	0.04	0.196	
Lobule V	−0.06	0.04	0.171		−0.03	0.04	0.477	
Lobule VI	−0.08	0.04	0.070		−0.08	0.04	0.032	^b^
Crus I	−0.20	0.08	0.010	^a^	−0.17	0.08	0.034	^b^
Crus II	−0.20	0.10	0.038	^b^	−0.21	0.10	0.048	^b^
Lobule VIIb	−0.15	0.05	0.005	^a^	−0.14	0.06	0.027	^b^
Lobule VIIIa	−0.11	0.04	0.005	^a^	−0.09	0.04	0.021	^b^
Lobule VIIIb	−0.09	0.04	0.019	^b^	−0.08	0.04	0.061	
Lobule IX	−0.14	0.07	0.051		−0.16	0.07	0.027	^b^
Lobule X	−0.18	0.12	0.117		−0.11	0.09	0.240	
Whole Cerebellum	−0.14	0.05	0.011	^a^	−0.13	0.05	0.016	^a^

*B* regression coefficient, *SE* standard error, *P* p-value.

^a^Indicates that the regional analysis passed correction for the false discovery rate <0.05.

^b^Indicates that the regional analysis was nominally significant but did not pass correction for the false discovery rate.

**Fig. 3:**
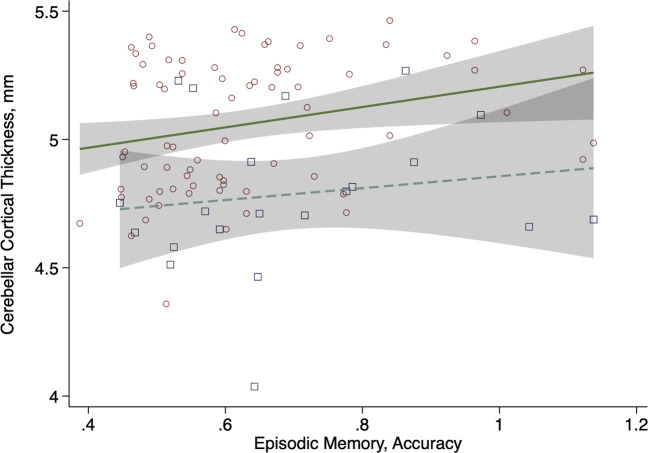
Association between cerebellar cortical thickness (mm) and episodic memory (coefficient = 0.35, standard error = 0.15, *p*-value = 0.021) adjusted for gender. Males: small red circles and solid green line; females: large blue squares and dashed line; 95% confidence intervals shown using transparent gray boxes.

Associations were also evident with cognitive performance, particularly episodic memory both with and without adjusting for sex (e.g., Fig. 3). Note that results were not significantly different between males and females, but the number of females in the sample does not allow for reliable subgroup analysis, showing only overall results. Results showed significant associations between episodic memory and cerebellar cortical thickness in Lobules VIIb, VIIIa, VIIIb, and IX. Analyses also showed associations between response speed and cerebellar cortical thickness in the Crus I and II (Appendix Fig. 1).

### Comparison with atrophy in neurodegenerative disorders

Interpretation of regional findings for reduced cerebellar cortical thickness in WTC responders with CI to known neuropathological conditions derived from results described in a meta-analysis of patterns of cerebellar atrophy shown in Table 3. Multi-region risk scores identified no clear signal indicating a single conditional etiology for WTC responders with CI, though orthogonalized results suggested that the overall cerebellar pattern was more indicative of disease state than disease-specific indices (Table 3).

**Table 3:** Diagnostic grouping as defined by regional differences in cerebellar atrophy.

	Model 1: Raw Scores		Model 2: Orthogonalized Scores	
Multi-Region Score	D	95% C.I.	AUC	95% C.I.
Mean cerebellar cortical thickness	0.68	0.58–0.77	0.68	0.58–0.77
Amyotrophic lateral sclerosis	0.68	0.57–0.79	0.45	0.35–0.56
Alzheimer’s disease	0.66	0.56–0.77	0.49	0.39–0.60
Multisystem atrophy	0.58	0.47–0.70	0.49	0.39–0.60
Progressive supranuclear palsy	0.65	0.54–0.76	0.49	0.38–0.59
Frontotemporal dementia	0.68	0.57–0.78	0.52	0.41–0.62

This table shows the overall capability for multi-region risk scores to accurately detect cognitive impairment.

*D* standardized mean difference, *AUC* area under the receiver operating curve, *95% C.I*. 95% confidence interval.

We also examined associations between cerebellar cortical thickness and physical functional impairments to find that cerebellar cortical thickness in lobule VIIIa was nominally associated with tandem balance difficulties (Appendix Fig. 2), with hemispheric results presenting as asymmetric with right-side reductions larger in both lobules.

Analyses examining differences between responders with/without PTSD identified no differences in the whole cerebellum (Appendix Fig. 3), nor in any cerebellar subregion even when focused on individuals with PTSD and CI when compared to those without or when examining the extent of PTSD symptomatology (results not shown). Additionally, analyses did not identify any association between high levels of WTC exposure and reduced cerebellar cortical thickness.

## Discussion

There remains a lack of clarity as to an accepted etiology for the early-onset CI observed in WTC responders, or for the presence of early-onset physical functioning decline associated with CI in this population. This study used an automated neuroimaging pipeline to characterize patterns of reduced cerebellar cortical thickness in WTC responders with CI. The primary goal of this study was to determine whether WTC responders with CI had patterns of reduced cortical thickness in the cerebellum and, if present, whether the pattern of reduced cortical thickness was consistent with a known neurodegenerative disorder. This study, described while considering results from a previous meta-analytic study (see Table 4 for a summary of those results), suggested that WTC responders with CI had reduced cerebellar cortical thickness evident in regions that did not match patterns for known neurodegenerative conditions.

**Table 4:** Interpretation of evidence in comparison to previously published meta-analysis.

Region	Functioning	Reduced thickness pattern	WTC
Lobule I/II	Motor, vestibular	MSA (R, L), PSP (L)	
Lobule III	Motor, vestibular	MSA (R, L), PSP (L)	
Lobule IV	Motor, vestibular		
Lobule V	Motor, somatosensory	ALS (R)	L
Lobule VI	Motor, language, spatial	ALS (L), AD (R)	L
Crus I	Language (R), working memory, executive function, affective	PSP (L), ALS (L), **AD (*R*)**	***R***, L
Crus II	Language (R), working memory, executive function, affective	AD (R), FTD (R, L), PSP (L), ALS (L)	S
Lobule VIIb	Executive, language, affective	**FTD (*R****,* L), PSP (L)	***R****,* L
Lobule VIIIa	Motor, working memory, language	**ALS (R)**	***R***, L
Lobule VIIIb	Motor, somatosensory	ALS (R)	R, L
Lobule IX	Visuomotor, memory, affective	PSP (R)	L
Lobule X	Vestibular		

Hemispheric results are listed using L for left and R for right. Boldface cortical thickness patterns denote results that overlap with WTC-related regions shown in the rightmost column. WTC patterns that were nominally significant are listed, while regions that passed the false discovery rate.

*MSA* multiple system atrophy, *PSP* progressive supranuclear palsy, *ALS* amyotrophic lateral sclerosis, *AD* Alzheimer’s disease, *FTD* frontotemporal dementia, *WTC* World Trade Center responder.

We identified associations between reduced cerebellar cortical thickness and poorer episodic memory as well as slower response speed. Reasons for this finding might include that atrophy in the cerebellum is concurrent with neurodegeneration elsewhere in the brain. Indeed, while often conceptualized as different systems, cognitive and physical functioning are related as individuals age and correspondence is most consistent when examining measures of cognitive and physical functioning such as response speed and handgrip strength that requires coordination and finger or arm motion [36]. It is possible that these results are emerging because the cerebellum is showing similar results to those seen elsewhere in the brain.

An alternative theory may be that in acting as a relay station between other parts of the brain, the cerebellum plays a critical functional role in maintaining cognition [4]. There is increasing evidence from functional connectivity analyses show stimuli can impact both neocortical and cerebellar regions are activated for different cognitive functions including motor functioning, language comprehension, spatial reasoning, and working memory [37]. Physical and cognitive decline has been linked to disrupted hippocampus-amygdala-cerebellum connections, consistent with reduced cerebellar gray matter volume associated with lower delayed recall, visuospatial, executive, and verbal fluency scores [38]. In our study, episodic memory was associated with cerebellar cortical thickness in Lobules VIIb, VIIIa, VIIIb, and IX. These findings correspond to results in both working memory and language identified on functional imaging [39]. Analyses also showed associations between response speed and cerebellar cortical thickness in the Crus I and II, in accordance with prior functional neuroimaging analyses of working memory [39], and findings that have linked response speed performance to morphological changes in these regions [40]. Further multimodal work examining the cortical correspondence and linking white matter tractography with functional neuroimaging may help to understand these questions.

Prior studies have suggested that the responders might be experiencing ADRD. For example, one study reported biomarkers indicative of the amyloid, tau, and neurodegeneration (ATN) cascade of AD [41] when using plasma biomarkers collected in a sample of 398 WTC responders with and without CI [42]. Another study noted that reduced cerebellar cortical thickness in the cerebrum of WTC responders with CI presented similarly to an early-onset parietal-dominant AD subtype [9, 43]. However, this study suggested that CI in WTC responders was not clearly a result of any known disease process including AD. An alternative hypothesis is that the present sample of WTC responders with CI may be experiencing a parietal-dominant form of AD [43], which is an early-onset subtype of AD that is not well understood. At the time of this writing and to the best of our knowledge, volumetric cerebellar data in patient populations with parietal-dominant AD were not available for comparative analysis. Future neuroimaging studies with WTC responders experiencing early-onset CI should seek to address this.

Definitive evidence supporting or negating the presence of ADRD in WTC responders with early-onset CI will be generated from molecular neuroimaging analyses using amyloid and tau positron emission tomography (PET) ligands. Yet, the present results suggest that these analyses should be aware of the potential for cerebellar cortical atrophy to affect results on PET, since the cerebellum and the cerebellar cortical matter, are often used as reference regions in both amyloid and tau imaging [44]. To circumvent this, future work in this population should determine whether there is a need to rely on the cerebellar white matter as a reference region for molecular brain imaging, as potential atrophy in the cerebellum of WTC responders with CI could lead to miscalculations of PET ligand uptake values in other ROIs. Therefore, future neuroimaging studies with PET ligands in the WTC population should explore alternative brain regions such as the cerebellar white matter to be used as a reference region.

WTC responders are at elevated risk of experiencing CI, and the presence of PTSD has emerged as a central risk factor [45]. In this study, we could not identify differences between responders with and without PTSD, and analyses also did not identify associations between the extent of reduced cerebellar cortical thickness and PTSD diagnosis or with symptom severity. The lack of identifying such associations may be due to the small sample size. Therefore, future neuroimaging studies with larger WTC responder samples should interrogate this question further. Our present findings match those from prior studies in the WTC population showing that brain volumetry in responders is associated with the presence of CI, independent of the presence of PTSD [14, 43]. Considering the evidence that the presence of PTSD emerged as a central risk factor for CI in WTC responders, these results are somewhat perplexing as they do not clearly demarcate the process by which PTSD in WTC responders might cause elevated risk to a neurodegenerative condition. Recent studies have tried to understand this process using serologic biomarkers to show that both Neurocan and Brevican proteins are present in responders with PTSD and CI, potentially indicating the potential for intraneuronal loss that may exacerbate other neurodegenerative processes [46]. Therefore, future studies with WTC responders interrogating serologic biomarkers should consider investigating proteomic levels. However, alternative theories could suggest that the neuroimmune system might cause monocytic and macrophagic dysregulation in those who have been heavily exposed to NPM at the WTC [47, 48]. Further work may help to improve our understanding of the role of PTSD in increasing vulnerability to neurodegenerative disease in WTC responders.

### Limitations

This study is limited in several ways including the small sample size and the lack of individuals with MCI. Additionally, while we over-sampled racial/ethnic minorities and women, this study still has a relatively small number of both making stratified analyses challenging. The generalizability of the results from this study to other populations is limited by the shared occupations and exposures of the source population. This is a cross-sectional study and, therefore, we cannot presently ascertain whether reduced cerebellar cortical thickness is a result of a neurodegenerative process or due to anatomical differences that preceded the WTC exposures. We also cannot determine if the results shown here are a cause or consequence of CI. Additionally, we cannot determine whether reduced cerebellar cortical thickness is progressive or emerged instead from a single insult and, therefore, we cannot determine the prognosis for findings of reduced cerebellar cortical thickness here.

## Conclusion

Prior evidence is suggesting that WTC responders may be experiencing a heightened risk of early-onset ADRD [16], though the etiology and prognosis of this emerging condition remain unclear. In this novel study, we examined the extent of reduced cerebellar cortical thickness in WTC responders with CI, who fit diagnostic criteria consistent with multidomain CI, as compared to those who were cognitively unimpaired. We found that responders with CI had evidence of reduced cerebellar cortical thickness that was significant in nine of the twelve bilateral lobules and in the whole cerebellum. Furthermore, reduced cerebellar cortical thickness was overlappingly present across lobules commonly atrophied in known neurodegenerative diseases, such as AD, but with no signal discovered to match one of these neuropathological conditions. In the context of epidemiological data, we conclude that these results may indicate the presence of encephalopathy of an unknown etiology potentially relating to their exposures on-site, although further work is needed to improve the characterization of such a novel condition.

## Supplementary information

Supplemental Appendix

## Data Availability

Medical information is protected, so only processed de-identified data will be made available upon receipt of a written request to the corresponding author. Raw image files may be accessed via a data use agreement.

## Code availability

The CERES pipeline used here to calculate cerebellar cortical thickness is publicly accessible online at www.volbrain.net.
