# Supplementary material for: Reduced cerebellar cortical thickness in World Trade Center responders with cognitive impairment
Source: Transl Psychiatry. 2022 Mar 16;12:107. doi: 10.1038/s41398-022-01873-6 (PMC8927406; doi:10.1038/s41398-022-01873-6)
Supplement: Supplementary file 1 — Supplemental Appendix [file 41398_2022_1873_MOESM1_ESM.docx]

**Appendix**

**Appendix Figure 1.** Standardized mean differences between cerebellar cortical thickness stratified by subregion and domain-specific cognitive functioning.

**Note:** Analyses adjust for sex and race/ethnicity. Numbers shown in black have nominal p-values below 0.10, while numbers shown in bold face were statistically significant, and numbers italicized in bold were statistically significant after accounting for the false discovery rate.

**Appendix Figure 2.** Standardized mean differences showing association between measures of physical functioning and cerebellar cortical thickness stratified by lobule

**Note:** Numbers shown in black have nominal p-values below 0.10, while numbers shown in bold face were nominally statistically significant, and numbers italicized in bold were statistically significant. None of the results passed adjustment for the false discovery rate.

**Appendix Figure 3**. Group mean marginal estimates of age/sex/post-traumatic stress disorder/intracranial volume-adjusted cerebellar cortical thickness (mm), stratified into cognitively unimpaired without PTSD (CU), post-traumatic stress disorder without cognitive impairment (PTSD), cognitive impairment without PTSD (CI), and CI with PTSD. P-values from generalized linear models adjusting for gender are shown.
